# 331. Emphasizing the O in OPAT: A Pathway for Ambulatory Outpatient Parenteral Antimicrobial Therapy (OPAT) Initiation from an Academic Infectious Diseases Clinic

**DOI:** 10.1093/ofid/ofad500.402

**Published:** 2023-11-27

**Authors:** Molly Hillenbrand, Michael E Yarrington, Jason Funaro, Jenny Shroba, Kristen Dicks

**Affiliations:** Duke University, Durham, North Carolina; Duke University Health System, Durham, North Carolina; Duke University Hospital, Durham, North Carolina; Duke University Hospital, Durham, North Carolina; Duke University Health System, Durham, North Carolina

## Abstract

**Background:**

In response to rising inpatient costs, health care systems are investigating mechanisms to avoid unnecessary admissions and reduce inpatient length of stay. Outpatient parenteral antimicrobial therapy (OPAT) provides a pathway for patients to receive intravenous antimicrobials at home or in a skilled nursing facility (SNF); however, admission is often required for OPAT initiation. Our interdisciplinary OPAT team developed a process to initiate OPAT directly from our infectious diseases (ID) clinic (Figure 1). We report our single-center experience and patient outcomes.

**Figure 1.**
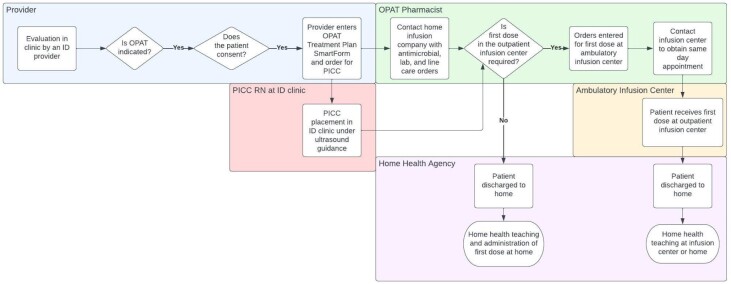
Process map for ambulatory outpatient parenteral antimicrobial therapy (OPAT) initiation. ID, Infectious diseases; PICC, peripherally inserted central catheter.

**Methods:**

We conducted a consecutive case series review of patients who completed OPAT episodes started directly from our ID clinic between July 1, 2022, and March 31, 2023. Patients with a history of solid organ transplant, hematologic malignancy, or end stage renal disease on hemodialysis are not managed by our formal OPAT team and were thus excluded. We reviewed patient demographics, clinical characteristics, and adverse events while receiving OPAT. Admission rates for inpatient and outpatient cohorts were compared using Pearson Chi Square analysis.

**Results:**

Thirty-seven patients were started on OPAT directly from our ID clinic during the study period (Table 1, Figure 2). Most patients had intravenous access placed by a vascular access nurse in our clinic, and all received OPAT at home. The 30-day admission rate for this cohort was significantly less than OPAT patients who were discharged from the hospital during the study period (157/684 vs 1/36, p=0.004). One patient in this series was admitted to the hospital for persistent prosthetic joint infection requiring repeat surgical intervention. Serious OPAT-related adverse events in this cohort were less than previously published rates (Table 2), and line complications were addressed in our clinic in 3/6 cases, avoiding emergency department visits.

**Table 1.**
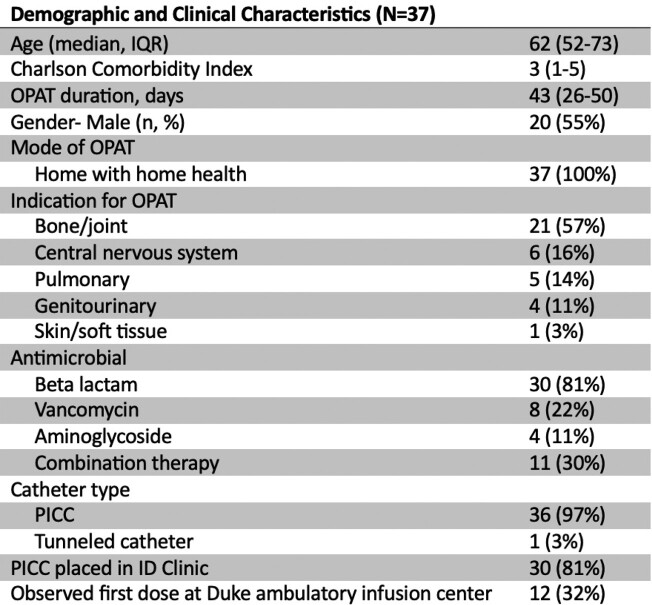
Demographic and clinical characteristics of 37 patients started on OPAT directly from ID clinic. IQR, interquartile range; PICC, peripherally inserted central catheter.

**Figure 2.**
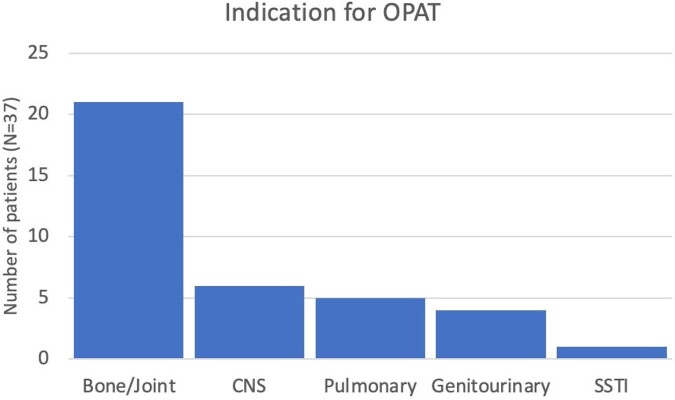
Indications for OPAT in 37 patients started on OPAT directly from ID clinic. CNS, central nervous system; SSTI, skin and soft tissue infection.

**Table 2.**
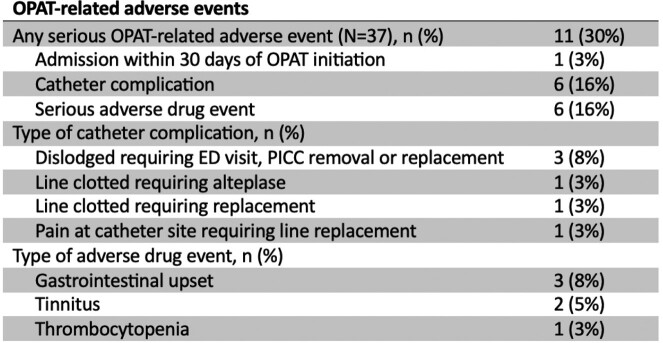
OPAT-related adverse events in 37 patients started on OPAT directly from ID clinic.

**Conclusion:**

This case series demonstrates that OPAT initiation directly from an ID clinic is feasible and safe, with lower admission and complication rates than patients discharged from acute care settings on OPAT. A robust ambulatory OPAT program with an interdisciplinary OPAT team can result in substantial cost savings for healthcare systems.

**Disclosures:**

**Kristen Dicks, MD**, UpToDate: Advisor/Consultant

